# Case Report: Unique immunotherapy response in a patient with metachronous colorectal cancer not associated with Lynch Syndrome

**DOI:** 10.3389/fonc.2025.1648320

**Published:** 2025-10-15

**Authors:** Nithya Krishnamurthy, Deirdre Cohen

**Affiliations:** ^1^ Icahn School of Medicine at Mount Sinai, New York, NY, United States; ^2^ Mount Sinai Health System, Department of Hematology and Oncology, New York, NY, United States

**Keywords:** metachronous colon cancer, pembrolizumab, immunotherapy response, MSI-high, colon cancer

## Abstract

Metachronous colorectal cancers (mCRC) occur in ~3.4% of cases within 10 years of initial diagnosis, with risks elevated in hereditary conditions like Lynch syndrome. We report a case of a 78-year-old male with a history of left-sided colon cancer (pT2N0M) resected in 2015 without adjuvant therapy, presenting in 2024 with a proximal ascending colon mass. The initial tumor was poorly differentiated adenocarcinoma, MLH1 and PMS2-deficient, and exhibited BRAF overexpression. The metachronous tumor was a moderately differentiated adenocarcinoma with a tumor mutational burden of 58 mutations/megabase and a BRAF V600E mutation. At the time of the second colon cancer diagnosis, germline testing was negative for Lynch syndrome, and Pembrolizumab was initiated due to the mismatch repair-deficient (MMR) status. The patient had a remarkable response to immunotherapy, with complete resolution of the colonic tumor on subsequent colonoscopies 3 and 6 months after initiation of immunotherapy with single-agent Pembrolizumab. Despite the absence of familial predisposition, microsatellite instability high (MSI-H) and MMR-deficient tumors confer increased mCRC risk. Surveillance remains critical post-resection, particularly in patients with MSI-H and MMR-deficient tumors, even without Lynch syndrome. Further studies are needed to elucidate mCRC risks and outcomes in non-Lynch syndrome, MSI-H colorectal cancer cohorts.

## Introduction

Colorectal cancer (CRC) is the third most frequently diagnosed cancer worldwide. In the U.S., an estimated 154,270 new cases and 52,900 deaths are expected in 2025 (1, 2). Advances in CRC treatments and early detection have significantly improved survival rates, leading to a growing population of CRC survivors. However, this progress has also increased the risk of developing metachronous CRC—a new primary tumor occurring after an initial diagnosis. The risk of metachronous CRC within five years of curative surgery ranges from 2% to 12% (3).

Hereditary CRC syndromes are major contributors to metachronous CRC (mCRC), increasing the risk of a second tumor by 10-20% (4, 5). Among these, Lynch syndrome is the most prevalent, with a metachronous tumor rate of 12-33% over a follow-up period of up to 15 years (6). This risk can be significantly reduced to 0-6% with subtotal or total colectomy (6).

Additionally, a family history of CRC is associated with an elevated risk of metachronous neoplasia, as are microsatellite instability-high (MSI-H) or mismatch repair (MMR)-deficient primary CRCs (7, 8). MMR deficiency or MSI-H occurs in approximately 15% of CRC cases and can arise sporadically, independent of hereditary syndromes such as Lynch syndrome (9). MLH1 and PMS2 deficiencies are hallmark features of Lynch syndrome (10), with only rare instances of PMS2 loss of expression reported, typically resulting from MLH1 gene hypermethylation or somatic BRAF V600 mutations (11).

Our case illustrates an instance of non-Lynch syndrome metachronous MMR deficient/MSI high colon cancer diagnosed nine years apart, with an interval serrated adenoma found during colonoscopy surveillance.

There is no consensus on standardized colonoscopy surveillance protocols following surgery for CRC in patients without a hereditary CRC syndrome. Current recommendations generally suggest performing a colonoscopy one year after surgery, followed by subsequent colonoscopies at three and five years after the initial post-surgery colonoscopy. Continued surveillance every five years may also be considered based on individual risk factors and life expectancy (12).

## Case description

We describe the case of an otherwise healthy 78-year-old Caucasian male with a past medical history of left-sided colon cancer diagnosed in 2015 during a routine screening colonoscopy. He has no family history of colon, endometrial or ovarian cancer. He underwent a left hemicolectomy in 2015, complicated by an anastomotic leak that required bowel resection and a colostomy which was reversed later that year. Pathology revealed invasive, poorly differentiated colon adenocarcinoma. The tumor measured 4 cm at its greatest dimension and invaded into, but not through, the muscularis propria. Tumor necrosis and prominent tumor-infiltrating lymphocytes were noted. Lymphovascular invasion was present. Examination of 15 lymph nodes showed no evidence of tumor involvement (0/15). The tumor was staged according to the AJCC classification as pT2N0Mx. Additional findings included multiple hyperplastic polyps. All resection margins were free of tumor. He did not receive any adjuvant therapy and in concordance with NCCN guidelines, a colonoscopy and CT Chest Abdomen Pelvis one-year post-surgery was done without any recurrent disease or polyps (13). A subsequent colonoscopy in 2022 revealed a 1cm serrated adenoma in the ascending colon which was entirely removed.

**Figure 1:**
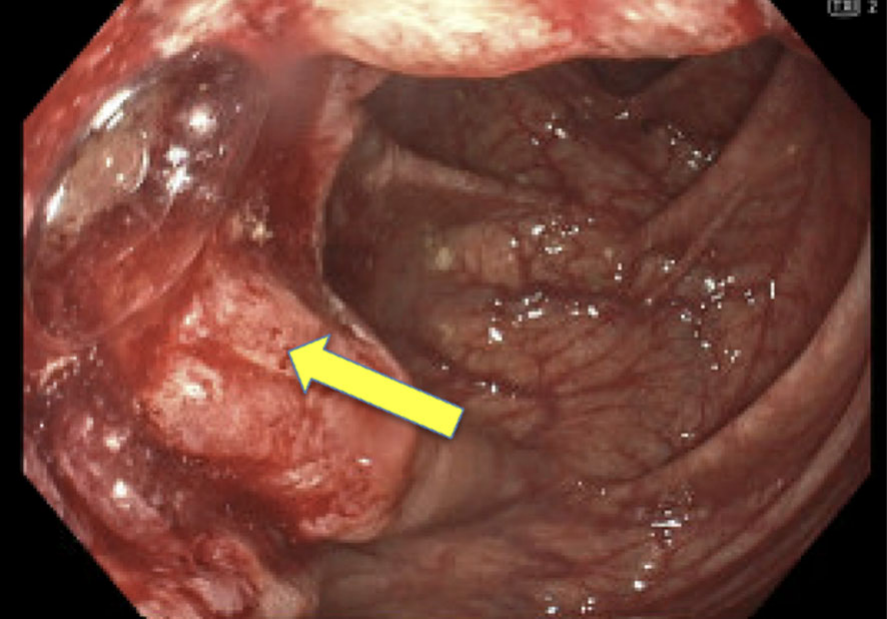
Pre-Treatment. Polypoid non-obstructing 4 cm mass in the proximal ascending colon with tattoo placed on opposite wall from mass in May 2024.

In May 2023, the patient had a colonoscopy procedure that was reported as normal, but due to poor preparation, a repeat colonoscopy was conducted in 2024. In May 2024, this repeat colonoscopy revealed a polypoid 4 cm non-obstructing mass in the proximal ascending colon (**Figure 1**). The patient had a normal physical exam and serum chemistries. Complete blood count demonstrated a very mild normocytic anemia. The cancer was staged using colonoscopy and CT chest abdomen pelvis. Findings supported a clinical stage of cTxN0M0, indicating a localized tumor with no nodal or metastatic disease. Pathology was consistent with an invasive moderately differentiated adenocarcinoma.

The patient’s primary tumor in the left colon in 2015 was an invasive poorly differentiated adenocarcinoma with MLH1 and PMS loss, MSH2 and MSH6 retained, and BRAF overexpression. The metachronous proximal colon tumor was an invasive moderately differentiated adenocarcinoma also with MLH1 and PMS2 loss and retained MSH2 and MSH6 in tumor cells. TMB was found to be 58 muts/megabase. Genomic profiling of the tumor revealed mutation in BRAF V600E. The patient was tested for germline mutations in a 70 gene multi-cancer panel including genes associated with Lynch syndrome (MLH1, MSH2, MSH6, PMS2, EPCAM) which were found to be negative. Of note, patient was found to have a germline BRCA2:c:8351 variant of unknown significance with conflicting data regarding pathogenicity which differed from the somatic variant BRCA2 T2685 fs*9.

**Figure 2:**
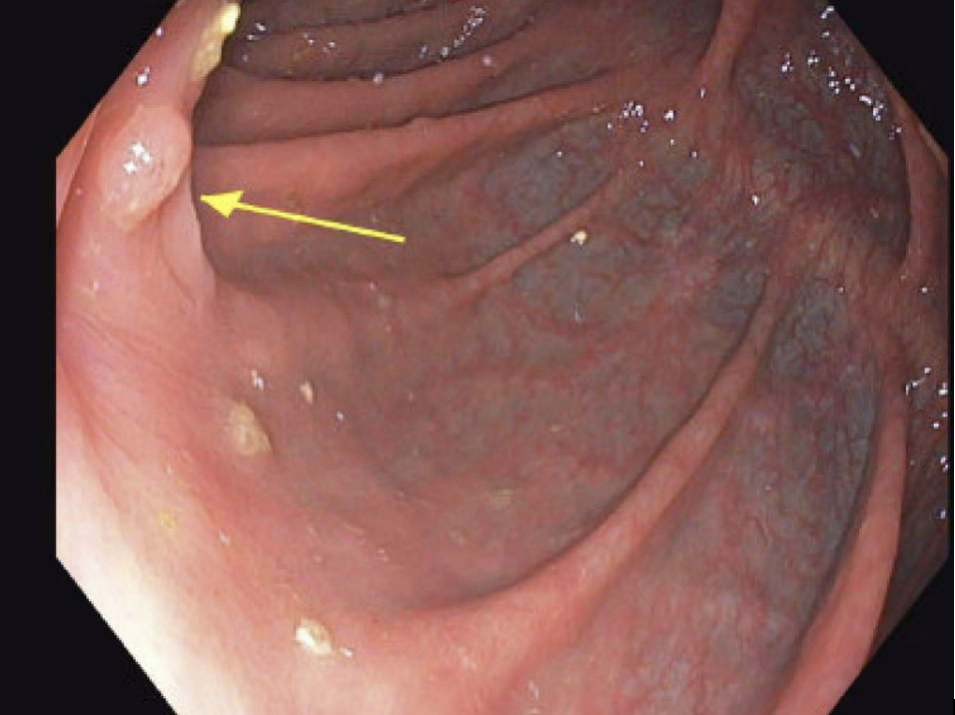
Post-Treatment. December 2024 colonoscopy. No mass was evident in the right colon; the previously involved folds opposite the tattoo showed only mild granularity and a small 4 mm nodular area, now nearly completely normalized compared to the May and August 2024 examinations.

Due to the patient’s prior complications from initial colon surgery, personal wishes, MMRd tumor and high functional capacity, single-agent pembrolizumab was initiated. Through a shared decision-making process, the patient was thoroughly informed of the risks, benefits, and available alternatives, including the standard of care. It was emphasized that a watch-and-wait approach, specifically forgoing surgery, is not considered standard of care. The decision was made following multidisciplinary consultation, and the patient provided informed consent for this approach, expressing a preference to avoid both surgery and chemotherapy. Notably, after three cycles, colonoscopy demonstrated near-complete resolution of the tumor. Following a second series of six infusions, complete resolution was confirmed on colonoscopy, biopsy, and CT chest abdomen pelvis in December 2024 (See **Figures 1** and **2**). Pathology findings in August demonstrated colonic mucosa with mild post-inflammatory changes and no tumor detected, and in December noted colonic mucosa with lymphoid aggregates, mildly active nonspecific colitis, and no tumor was seen. The patient’s follow up monitoring strategy includes serial colonoscopies and CT imaging every 6 months for 5 years.

## Discussion

Metachronous colorectal cancers, which are secondary tumors occurring in the same patient after an initial colorectal cancer diagnosis, were predominantly detected 3 to 5 years following the initial surgery (14). Recent studies report a 3.4% risk of multiple primary colorectal cancers within 10 years of the initial diagnosis of all colorectal cancers (15). Other studies have put the risk of metachronous CRC within five years of curative surgery from 2% to 12%.

Of note, patients with a family history of colorectal cancer, associated colorectal adenomas, or a history of malignant tumors in other organs are at increased risk for developing metachronous multiple colorectal carcinomas (16). This increased prevalence may be linked to hereditary conditions such as Lynch Syndrome; the risk of metachronous colon cancer in patients with Lynch syndrome was found to be 22.8% in those with segmental resection versus 6% in those with extended colectomy (17).

Interestingly, our patient tested negative for germline mutations related to Lynch syndrome and had no family history of colorectal cancer or inflammatory bowel disease, which could increase the incidence of metachronous colon cancer. He did have two MMR-deficient colon tumors, which are associated with a higher likelihood of metachronous colon cancer (18–20). In a global study of colorectal cancer cases where those with Lynch syndrome or MUTYH mutation carriers were excluded, 138/6085 (2.3%) of colorectal cancer cases were found to have a metachronous colon cancer over a median follow-up of 12 years. Interestingly, those with MMR-deficient tumors in this non-Lynch cohort were found to have a 72% increased risk of metachronous colorectal cancer compared to those with MMR-proficient tumors (21). Notably, our patient had a sessile serrated adenoma in 2022. The serrated pathway in colorectal cancer is characterized by early BRAF V600E mutations, which activate the MAPK-ERK pathway and drive tumor progression. Serrated tumors with MSI exhibit faster progression, and BRAF mutations are common in sessile serrated adenomas but rare in conventional adenomas, highlighting an alternative route to colon carcinogenesis (22).

Other retrospective studies have looked at rates of metachronous colorectal cancer in patients without germline Lynch syndrome mutations. One retrospective study from Japan of patients with submucosal resection showed a 7.6% overall incidence rate on surveillance colonoscopies performed up to 5 years after initial primary cancer (23). In another retrospective analysis of 19,731 patients undergoing resection for colorectal cancer and excluding those with Lynch syndrome, 191 (1%) had metachronous colorectal cancer (24). Even as metachronous colorectal cancer is rare without a familial predisposition or Lynch syndrome diagnosis, it is important to maintain surveillance for colorectal cancer patient’s post-resection, especially in the setting of MSI high and/or MMR-D.

Remarkably, the patient achieved a complete response, with resolution of the tumor confirmed by colonoscopy after six cycles of single-agent pembrolizumab. Single-agent pembrolizumab has been shown to significantly prolong progression-free survival and, in updated analyses, to achieve comparable overall survival to chemotherapy when used as first-line therapy for MSI-H/MMRd metastatic colorectal cancer, with fewer treatment-related adverse events (25, 26). Additionally, a recent trial demonstrated a 44% pathological complete response (pCR) rate in 85 patients with early-stage (I–III) MSI-H/MMRd colorectal cancer following a single cycle of neoadjuvant pembrolizumab (27). In a separate phase 2 study, treatment with neoadjuvant nivolumab plus ipilimumab in 111 patients with locally advanced dMMR colon cancer resulted in a 68% pathological complete response rate and no recurrences at a median follow-up of 26 months (28).

Recently, Dostarlimab, another anti–PD-1 monoclonal antibody, demonstrated remarkable efficacy in a phase 2 trial of dMMR rectal cancer, with all 49 patients achieving a clinical complete response and avoiding chemoradiation or surgery (29). While these results highlight the transformative potential of immunotherapy in select subsets of colorectal cancer, the optimal duration of therapy, long-term outcomes, and the most effective strategies for surveillance remain undefined. Future clinical trials should address these knowledge gaps to guide evidence-based management and follow-up of patients managed nonoperatively.

Patient Perspective: “Before and after the beginning of my tumoral episode in May 2024, I have never felt very sick or debilitated and have always been able to carry out my regular responsibilities. I noted further that my levels of general fitness and well-being increased progressively throughout the two series of immunotherapy completed in November 2024. I feel that this success may be due to both the excellent care I received and my strong personal health resources”.

## Data Availability

The original contributions presented in the study are included in the article/Supplementary Material. Further inquiries can be directed to the corresponding author.
